# The author who wasn’t there? Fairness and attribution in publications following access to population biobanks

**DOI:** 10.1371/journal.pone.0194997

**Published:** 2018-03-23

**Authors:** Erika Kleiderman, Amy Pack, Pascal Borry, Ma’n Zawati

**Affiliations:** 1 Centre of Genomics and Policy, Department of Human Genetics, McGill University, Montreal, QC, Canada; 2 Department of Public Health and Primary Care, Centre for Biomedical Ethics and Law, University of Leuven, Leuven, Belgium; Institut Català de Paleoecologia Humana i Evolució Social (IPHES), SPAIN

## Abstract

We conducted a document analysis that explored publication ethics and authorship in the context of population biobanks from both a theoretical (e.g. normative documents) and practical (e.g. biobank-specific documentation) perspective. The aim was to provide an overview of the state of authorship attribution in population biobanks and attempt to fill the gap in discussions around the issue. Our findings demonstrate that the most common approach adopted in both the normative and biobank-specific documentation is acknowledgment. A co-authorship approach was second and highlighted concerns surrounding the fairness of imposing authorship of the scientific leadership as a condition to access data and biosamples, as well as the alignment with the International Committee of Medical Journal Editors’ criteria such as what is deemed a significant contribution and how to ensure accountability. Based on these findings, we propose a three-prong approach, that may be cumulative, to address the issue of authorship attribution in the context of population biobanks, namely 1) the biobank should be appropriately acknowledged; 2) an invitation for co-authorship should be made based on the spirit of collaboration and provided a substantial contribution has been made; and 3) a citation/referencing option should be available.

## Introduction

The maxim ‘publish or perish’ is well-known by academics and is an important factor that overlays any researcher’s career. Publications are one of the means of quantifying academic competence and are an essential component of evaluation for career advancement and a researcher’s assessment for funding [1]. While publishing is an important element, publishing in an ethically appropriate way is primordial. With increasing attention to research integrity, principles such as honesty, accountability, fairness, and openness are key pillars. Over the years, the question of who deserves authorship has been given much attention [2–8]. Furthermore, the evolution of research towards interdisciplinary approaches and increased collaboration, both nationally and internationally [9–13], has led to an increased number of authors on publications.

Authorship discussions are also present in the context of biobanking. In fact, one area that needs particular attention is the extent to which the scientific leadership of biobanks (i.e. individuals who hold a leadership role in the management and operations of the biobank) should be included as authors on publications led by external researchers who have used data and/or biosamples from the biobank. Some believe that the process of data collection should be rewarded with co-authorship on publications stemming from the use of the biobank’s data and biosamples [14], while others believe that the scientific leadership cannot be held accountable for the work that is being published since they have only collected or curated the data and biosamples, thereby not satisfying conditions for authorship [15–16].

A lack of recognition of the time and effort spent by a biobank’s scientific leadership, collecting and curating data and biosamples, can lead to an unwillingness on their part to share such materials. This would ultimately have a negative impact on the scientific community, as the efficiency of biobank research and sharing of data and biosamples would be compromised [17–18]. One attempt at addressing the lack of appropriate recognition of scientific contributions behind the creation and maintenance of a biobank has been the Bioresource Research Impact Factor (BRIF) initiative [19]. The BRIF initiative attempts to promote and facilitate data and biosample sharing by proposing a standardized citation format for biobanks within publications to recognize principal investigators [20–21]. However, the question at hand differs from the work done by this initiative, as it tackles authorship attribution, for which there is currently no clear guidance in the context of biobanks.

The purpose of this article is to shed light on authorship attribution in population biobanks and to try to fill the gap in discussions around authorship within biobank management and operations. Population biobanks are typically publicly funded entities considered to be a “public good contributing to the improvement of public health” (p. 725) [22]. The goal is to motivate sharing and encourage a wide range of research possibilities that may lead to potential benefits for the population at large, rather than for a specific disease. The public nature of these resources entails a commitment to share its data and biosamples, which in turn can be expected to lead to greater variety (interdisciplinarity) and productivity, more access requests, and result in greater output in the form of publications [23]. This ultimately justifies the importance of outlining authorship attribution and appropriate credit within the context of population biobanks in particular [23]. As a result, this becomes an interesting starting point to explore the issue of authorship attribution. Findings stemming from such research would remain easily adaptable to other types of biobanks as well. To our knowledge, no study has previously looked at this issue.

## Materials and methods

A document analysis was conducted with the aim of exploring the literature and guidance documents surrounding publication ethics and authorship in the context of data and biosample sharing, notably with regards to population biobanks [24–25]. Such an analysis “yields data that are then organized into major themes [and] categories […] specifically through content analysis” (p. 28) [26]. In order to do so, the situation was analyzed from a theoretical perspective, via normative documents related to human genetic research, to gain a better understanding of what the suggested approaches to authorship are and from a practical perspective, via biobank-specific documentation to better understand how authorship is being addressed by biobanks. The aim of this comparison was to capture similarities and differences between the two and to provide an overview of the state of authorship attribution in the context of population biobanks. The normative documents analyzed in this study primarily consisted of reports and guidelines (see S1 Table); while the biobank-specific documents generally consisted of the biobanks’ publication policies and guidelines, access policies and access agreements (see S2 Table). These documents were purposively selected because they are a primary source of information regarding publication norms. Such information can be found in detailed policies (e.g. publication policy), can be as simple as a paragraph inserted into the access policy, or can be found within or as part of the access agreement that legally binds the researcher and the biobank.

### Data sources

#### Normative documents

Normative documents were selected as an important source within this study owing to their probative force and, in the case of legislation, regulation and enforced guidelines, to their legally binding nature. These documents were found using *FULL TEXT* keywords such as ‘publication AND policy’, ‘authorship’, ‘acknowledgment’ in combination with the fixed *KEYWORD* ‘biobank’. The search date range was established from 2000 to 2017. A total of 17 normative documents consisting of reports and guidelines ranging from 2002 to 2015 were found applying this research strategy to the HumGen International Database (http://www.humgen.org), a database of national, regional, and international guidelines and policies specific to human genetic research.

**Fig 1:**
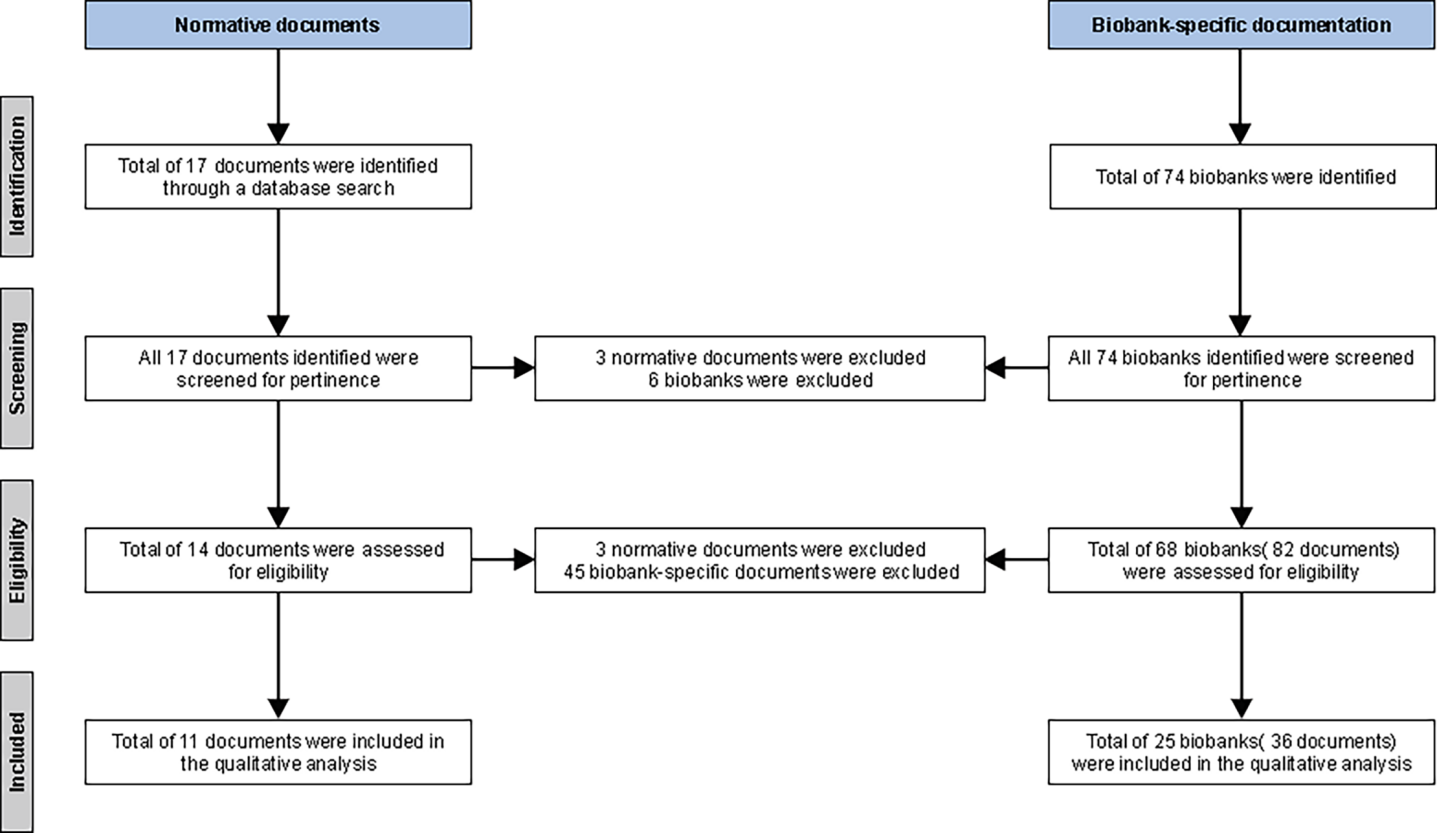
Document selection process.

Of these 17 documents, 3 were excluded for not meeting the pertinence criterion with regards to the mention of biobank authorship attribution. The 14 remaining documents were skimmed to ensure that each addressed categories that emerged from the literature (acknowledgment, co-authorship, citation/referencing). Only normative documents applicable to large-scale longitudinal biobanks were considered in order to align with the focus of this article and to allow for comparison with current biobank practices. Finally, of these documents, 11 respecting the aforementioned inclusion-exclusion criteria were deemed eligible for further appraisal. These remaining 11 documents were thoroughly analyzed in order to identify each organization’s recommendations and the existing mechanisms available to address biobank authorship attribution (Fig 1).

#### Biobank-specific documentation

The data collection process for biobank-specific documentation used a systematic list of 74 biobanks with publicly available access policies that appeared in a recent article by Langhof et al. [27] as a starting point for the identification and selection of population biobanks. These 74 biobanks were screened to identify longitudinal population biobanks that currently allow access to data or biosamples to external researchers and whose documentation was available in English or French. Disease- or condition-specific biobanks were excluded, as the focus of this manuscript is on authorship in the context of population biobanks. Only regional and national longitudinal biobanking initiatives offering data and biosamples for health and chronic illness research were eligible.

A total of 68 population biobanks were included. Eighty-two documents stemmed from the selected 68 biobanks as the search was not limited to biobank publication policies, but also included access policies, access agreements and data transfer agreements in order to complete or in lieu of an existing publication policy. These are the documents that address and include access and publication mechanisms and guidance.

The eligibility of these documents was then evaluated on the basis of pertinence with regards to biobank authorship attribution and categories derived from the literature review. After skimming through the documents, 36 documents stemming from 25 of the 68 biobanks were retained for analysis. These remaining documents were then thoroughly analyzed to pinpoint, for each biobank, both the approaches used and mechanisms in place to address the issue of authorship attribution.

### Data analysis

Document analysis was used to analyze the selected documents. Document analysis is a systematic method that retains aspects of both content and thematic analysis and facilitates the identification of relevant data to be analyzed. A deductive thematic approach was taken to advance the themes stemming from the literature: acknowledgment, co-authorship, and citation/referencing. For each concept-driven theme, sub-categories were identified and coded. For example, the sub-categories “requirements”, “contributions (significant)” and “automatic” were identified for the theme “co-authorship” (S1 File). The documents were initially coded by the first coder (EK), following which the second coder (AP) reviewed the proposed themes and sub-categories. Once both coders had gone through the documents, discrepancies in codes and sub-categories were discussed and refined until consensus was reached. This method allowed the identification of common patterns relating to the issue of biobank authorship attribution and comparison of these patterns across normative and biobank-specific documents.

Having access to final versions of currently implemented normative documents and biobank-specific documentation allows for the verification and confirmation of the organization’s current views on biobank authorship attribution.

## Results

Both the normative and biobank-specific documents were assessed in relation to categories that emerged from the literature. S1 Table presents a full list of the 11 normative documents that were included, as well as their associated recommendations. S2 Table contains a full list of the 25 included biobanks, their specific publication and access documentation (36 documents), along with their suggested approaches. In line with literature focusing on authorship and data sharing, the data was classified according to the following categories: acknowledgment, co-authorship, and citation/referencing. These topics served to organize the data to be analyzed. As mentioned above, the document analysis allowed us to compare and contrast theoretical (normative documents) and practical perspectives (biobank-specific documentation), enabling a better overview of what is being suggested and what is being done in practice regarding authorship attribution within this context.

### Acknowledgment

#### Normative documents

The majority of the normative documents analyzed (8/11) recommend an acknowledgment approach. More specifically, three types of acknowledgment were recomended: 1) biobank acknowledgment, 2) biobank curator acknowledgment, and 3) biobank and curator acknowledgment.

The first approach, consisting of biobank acknowledgment, is supported by the Organization for Economic Co-operation and Development (OECD). Indeed, the OECD 2009 *Guidelines* [28] recommend acknowledging the Human Biobanks and Genetic Research Databases (HBGRD) in publications and presentations that have used data and/or biosamples from HBGRD biobanks. This approach is also recommended by the International Society for Biological and Environmental Repositories (ISBER) *Best Practices for Repositories* [29], as well as the *Biobank Quality Standards* [30] produced by the National Cancer Research Institute (NCRI) and the Confederation of Cancer Biobanks (CCB). For example, the latter document states that “researchers should be required to acknowledge the biobank as the source of the [bio]samples used in their research” (p. 13) [30].

The second acknowledgment approach relates to biobank curators and is supported by five organizations. Biobank curator acknowledgment is in line with the Global Alliance for Genomics & Health’s general recommendation to acknowledge all who contributed to the results being published [31].

Similarly, Cancer Research UK, the Economic & Social Research Council (ESRC), the Medical Research Council (MRC), and the Wellcome Trust, in their joint report, emphasize the importance of ensuring appropriate acknowledgment of those who worked to produce the data. Specifically, the joint report encourages authors to consider the following position:

[…] it is usual to ask for undertakings on the nature of timing of research publications, and acknowledgments of data sources, designed to *protect the rights of the primary researchers responsible for generating the data* (the authors’ emphasis) [32].

Lastly, the third approach supports the acknowledgment of both curators and the biobank. Derived from an earlier MRC and Wellcome Trust report, it recommends that the acknowledgment of both the biobank curators and the biobanks always be included. Their 2006 *Report* [33] denotes that this necessary acknowledgment is usually placed in acknowledgment notes, either at the beginning or end of the publication. Similarly, the National Institutes of Health’s (NIH) *Genomic Data Sharing Policy* [34] requires the oral or written acknowledgment of both the NIH-designated data repositories and dataset by investigators using controlled-access and unrestricted-access data.

Contrary to these three approaches that clarify who should be acknowledged, RD-Connect does not specify whether the biobank or the curator should be acknowledged. They recommend, in their *International Charter of Principles for Sharing Bio-specimens and Data* [22], that the sharing of data and biosamples should follow criteria for the acknowledgment of intellectual contributions and originality based on the type of data available, for which they provide specific guidance by referring to rules of authorship and intellectual property rights.

#### Biobank-specific documentation

The majority of biobanks (17/25) have adopted an acknowledgment approach for biobank attribution. Within this majority, however, a few nuances may be discerned. The general approach is to acknowledge the biobank whose data and/or biosamples have been used to generate the results presented in the publication. This acknowledgment typically takes the form of a standard sentence or paragraph proposed by the biobank. For example, the UK Biobank’s *Access Procedures* clearly state that authors must acknowledge the biobank following the template: “This research has been conducted using the UK Biobank Resource” (p. 16) [35]. In addition to this template, 12 of the biobanks mention other requirements. More specifically, documents from the Avon Longitudinal Study of Parents and Children (ALSPAC), the Canadian Longitudinal Study on Aging (CLSA), and AMGEN, for example, require that the sources of funding be referenced.

Furthermore, it was noted that acknowledgment can become a secondary approach for biobanks if authorship cannot be attributed. This is the position taken by five of the biobanks–Canadian Partnership for Tomorrow Project (CPTP), CONSTANCES Cohort, Generation Scotland (GS), Million Women Study, and Marshfield Clinic Personalized Medicine Research Project (PMRP)–whose primary approach is co-authorship. These five biobanks also suggest an acknowledgment option either via a standard paragraph, reference to the methodological article, or inclusion of the biobank’s name in the publication title. For example, GS’s *Management*, *Access and Publications Policy* suggests a full acknowledgment of the biobank (template paragraph) if the standard criteria to merit authorship are not met (e.g. providing feedback on draft manuscripts and approval of the final version before submission).

Finally, it is worth noting that almost all biobanks (24/25) have some mechanism in place to monitor or review publications resulting from the use of data and/or biosamples directly integrated into their access procedures. These biobanks require principal investigators to provide a copy of the publication or presentation to the biobank once accepted. Of the 24 biobanks that employ tracking mechanisms, 14 (CLSA, CKB, EORTC, CONOR, HUNT Study, MoBa, BiB, GS, Million Women Study, ALSPAC, Newcastle Biomedicine Biobank, GUTS, Nurses’ Health Study, and EPIC) have a revision process in place, whereby a publication may not be submitted until it has been reviewed by the biobank. GS, ALSPAC, Nurses’ Health Study, and EPIC were the only biobanks to clearly state that approval is a requirement for journal submission. The review process for these 14 biobanks is primarily limited to ensuring that participants are not identified, that results are scientifically accurate, that the publication adheres to the biobank’s agreements, and that the publication would not bring the study into disrepute (see S2 Table). GS adds to its revision process the possibility of “assisting in the identification of patentable results,” “trying to identify overlap with other papers published or in preparation,” and ensuring that the biobank’s contribution is recognized (Appendix 11, *Generation Scotland Management*, *Access and Publications Policy*). The processing time for a biobank response typically ranges from one to two weeks from receipt of the publication. GUTS is an outlier, with a review process that takes at least one month. Six of the biobanks (CKB, EORTC, CONOR, BiB, GS and ALSPAC) also foresee the possibility of providing advice and feedback, when deemed useful; such feedback would not constitute formal peer-review.

### Co-authorship

#### Normative documents

None of the organizations analyzed in this study recommend attributing automatic co-authorship to the biobank’s scientific leadership. The *EAGDA Report*: *Governance of Data Access* set forth by Cancer Research UK, ESRC, MRC and Wellcome Trust clearly states that co-authorship should recognize significant contributions to a publication and not be a default requirement for permitting access to data. In the case of biobank staff members actively participating in the published research project, the International Society for Biological and Environmental Repositories’ (ISBER) *Best Practices* encourages the consideration of co-authorship. For co-authorship to be justified, “substantial intellectual input beyond the routine role of the repository” (p. 87) [29] must have been provided.

#### Biobank-specific documentation

Only the documentation of eight biobanks promote co-authorship as a primary approach (8/25): CPTP, CONSTANCES Cohort, GS, Million Women Study, Growing Up Today Study (GUTS), PMRP, Nurses’ Health Study, and the European Prospective Investigation into Cancer and Nutrition Biobank (EPIC). Five of the eight biobanks suggest that, where appropriate, at least one member of the biobank team either must or should be credited as co-author or offered co-authorship on publications arising from the use of the biobank’s data and/or biosamples. However, they also specify that substantial contributions to the development and management of the cohort must have been made and that all co-authors should have reviewed and approved any manuscripts. Only CPTP explicitly stipulates the need for all co-authors to meet the International Committee of Medical Journal Editors’ (ICMJE) criteria. Two of the biobanks (Million Women Study and PMRP) encourage co-authorship without providing further detail or articulating the level of contribution required (i.e. it is a way of recognizing the contributions of past and present staff and collaborators).

More detailed requirements for authorship attribution are outlined by both GS and EPIC. GS’ *Publication Policy* applies to both internal and external publications. Internal publications are written by members of the GS Executive Committee and the Expert Working Groups. External publications are written by collaborators having access to the GS resource. Although GS promotes a relatively inclusive view of authorship, it may only be merited if the following criteria are met: a) academic contribution to the design of the study, collection of data and analysis / reporting (i.e., “both intellectual responsibility and substantive work”); b) provide critical revision; and c) able to defend the paper as a whole. As mentioned in the previous section, if the above criteria are not met by an individual, only an acknowledgment of GS will be given (based on a standard paragraph).

EPIC documentation promotes automatic authorship in accordance with specific rules. It distinguishes its criteria based on whether the paper reports solely on EPIC materials and data or whether it is a consortium-based paper (i.e. materials/data from several studies). In the first scenario, where only materials or data from EPIC are used, authorship will be attributed in the following manner: writing group members; representatives of individual centres (grouped by country, in alphabetical order, according to the agreed number of authors per centre); the International Agency for Research on Cancer (IARC); the Imperial College London (ICL); and senior author (if applicable). In the second scenario, where materials or data from several studies, including EPIC, are used, authorship will be attributed in the following way: potential writing group members and EPIC representatives (up to 12 authors–one per country + one author from IARC + one author from Imperial College centres). EPIC outlines one exclusion criteria for both scenarios: if a country has not contributed data, collaborators from the relevant centre will not be included in the author list. However, exceptions may be considered based on individual contributions.

Furthermore, of the 18 biobanks whose primary approach is acknowledgment, six foresee the possibility for co-authorship according to appropriate conditions. This typically entails compliance with the ICMJE guidelines (i.e. all co-authors have significantly contributed and can take responsibility for the content of the publication). The CKB proposes that the list of co-authors end with “on behalf of” the biobank group. If the necessary criteria are not met, however, then an acknowledgment or contributorship statement is considered most appropriate.

### Citation/Referencing

#### Normative documents

In contrast to acknowledgment, the citation/referencing approach generally involves coding biobanks or researchers with unique identifiers and using such codes in publications to systematically track contributions. Three organizations encourage the citation approach: the European Commission, the Italian Society of Human Genetics, and the BRIF initiative. The European Commission proposes attributing unique identifiers to the biobank, to the researcher (using ORCID ID) or to the bioresource (using BRIF) [36]. Recommending the citation of only two of these three targets, the Italian Society of Human Genetics encourages authors to cite the origin of the biosample used and to quote the biobank in the publication.

The BRIF initiative, for its part, criticizes citing within a “bioresource field” or a specific section and even within the *Acknowledgment section* considering the necessity of traceability and easy retrieval. It goes on to discourage the identification of a bioresource using the name of the bioresource because it may create confusion. Instead, and along similar lines to the organizations mentioned previously, the BRIF initiative recommends identifying only the bioresource using BRIF identifiers (i.e. persistent, globally unique, citable and easily retrievable) and following the standardized citation of bioresources in journal articles (CoBRA) [20]. More specifically, it proposes that the citation be placed in the *Methods* section of the publication provided that the reference corresponds to a cited reference.

#### Biobank-specific documentation

None of the documents reviewed mentions the possibility of using citation or referencing as a primary approach for recognizing the efforts of biobanks and scientific leadership with respect to data collection. However, six biobanks adopting an acknowledgment approach also mention citation or referencing as a strategy for ensuring the visibility of their work. CLSA and the 1958 British Birth Cohort Study require that the core team responsible for the creation, collection, and implementation of the platform and data be referenced. Similarly, the UK Biobank and the China Kadoorie Biobank (CKB) request that the acknowledgment be linked to reference search engines such as PubMed or MEDLINE, where possible. Several biobanks, such as the Cohort of Norway (CONOR) and Born in Bradford (BiB), also add that the biobank’s relevant methodological article should be cited within the publication’s ‘Methods’ section. That said, four of the biobanks (HUNT, BiB, GS and ALSPAC) do require the inclusion of the biobank’s name as a keyword in any resulting publications.

## Discussion

Our analysis showed that the most popular approach to publishing using data and biosamples from population biobanks, in both normative documents and in practice, is that of acknowledgment. An acknowledgment approach allows for the appropriate recognition of those who have contributed to the data collection and curation.

A few caveats are relevant when discussing the potential co-authorship that results from accessing data resources. First, imposing authorship as a condition for access could potentially deter researchers from using biobanks. Second, such an imposition conflicts with certain recent normative documents, such as the UK Expert Advisory Group on Data Access’ report entitled *Governance of Data Access* in June 2015, in which good practice measures are outlined [32]. Of most interest and relevance to this topic is their guidance on establishing fair conditions for access. They highlight that those individuals who have been involved in the collection and curation of data should be duly recognized and credited for their efforts in the most appropriate manner [32]. They also affirm the notion that “co-authorship should [recognize] significant contributions to a publication and not be a default requirement for permitting access to data” (p. 10) [32]. Third, automatic authorship would be contrary to concepts of fairness (e.g. what happens if a scientific leader leaves the biobank and is not named, while a new leader, who did no work on the collection, is named in their place?) and accountability (e.g. the scientific leader will not necessarily review the article because reinforcing the standard conditions for authorship attribution is a challenge). Fairness suggests the need to highlight and acknowledge those who have significantly contributed to research and commands transparency, while accountability promotes quality assurance as well as the accuracy and integrity of the published article. Therefore, if co-authorship is possible, but not automatic, both fairness and accountability would be more likely to be respected *a priori*.

Although, citation/referencing (e.g. BRIF) pushes biobanks to improve and to ensure that they have a higher impact factor or visibility; it is a form of objective competitiveness. This approach aims to provide a way of tracking and referencing a biobank, yet it does not resolve the issue of authorship attribution, especially when the biobank’s scientific leadership have worked hard to collect and prepare the data.

However, in the current context, scientific journals typically refer to the ICMJE criteria when it comes to authorship attribution, which has undergone its own fair share of revisions since it was first published in 1978 under the name “Uniform Requirements for Manuscripts Submitted to Biomedical Journals” [37]. The following four cumulative ICMJE criteria are considered to be the internationally accepted standard for authorship attribution:

1. substantial contributions to the conception or design of the work; or the acquisition, analysis, or interpretation of data for the work; 2) drafting the work or revising it critically for important intellectual content; 3) final approval of the version to be published; and 4) agreement to be accountable for all aspects of the work in ensuring that questions to the accuracy or integrity of any part of the work are appropriately investigated and resolved [38].

The last two criteria posited by the ICMJE are reasonably adaptable to population biobanks. That said, the first two would require much more nuance and clarity, for what is meant by “substantial” is not always clear. Would the meaning of “substantial contribution” change depending on the type of data or biosamples involved? Irrespective of this, the way authorship is defined and how these criteria for authorship attribution are used, varies from one domain to another [39]. As such, there has been a recent push to develop an “alternative method of crediting biomaterial contributors, to ensure appropriate authorship inclusion and promote collaborative research involving biobanks” (p. 1) [16]. Authorship entails playing a substantial role in the conception and production of the publication, and involves “owning a stake in the product, where those listed as authors understand the final product, can defend and explain the final product, and endorse the final product.” As such, authorship can be qualified as the “*sine qua non* for the paper or project, indicating a fundamental element of the whole” (p. 6) [40]. However, criticisms surrounding the ICMJE authorship criteria have been brought forth by several authors. For example, the lack of an explicit requirement for authors to have been intellectually involved in the research, and the inability—especially in interdisciplinary publications—for all researchers to have a complete understanding of all the details of the work [41]. In other words, while the ICMJE criteria refer to important intellectual content regarding the drafting of the manuscript; it remains silent as to involvement in the research.

Based on this, we propose the following approach, which may be a cumulative process, to address the issue of authorship attribution in the context of population biobanks:

1. Acknowledgment of the biobank as the source of the data and biosamples used to conduct the research. Biobanks should consider creating a standard clause that would be added to a “Publications Policy,” an “Access Policy” or an “Access Agreement”.
2. Depending on the type of biobank (e.g. population biobank) and the type of data and/or biosamples (e.g. blood, saliva, urine) that are requested, an invitation for co-authorship could be suggested. This does not comprise automatic authorship attribution, but is simply an invitation to be named a co-author in the spirit of collaboration and fairness. Such principles are important, but may be difficult to impose in a policy. The invitation should be supported by a system of coordination, especially if there are a large number of scientific leaders. Communication between the scientific leadership and approved users (i.e. researchers) should be facilitated. Authorship will only be bestowed if the following criteria are met: a) substantial contribution in the preparation of the materials being accessed, and b) substantial contribution to the drafting of the article and review of the final draft (these should be cumulative). Population biobanks should also list the scientific leadership that could be invited, along with their general expertise, on their websites so that researchers interested in gaining access to data and biosamples are aware of the key players.
3. There should also be a citation/referencing option available (e.g. CoBRA), where population biobanks will have to choose one of the options mentioned in the *Guideline to Standardize the Citation of Bioresources in Journal Articles*, such as using an ID, a consolidated acronym or referencing the biobank’s marker paper [20]. This will improve the biobank’s traceability and the quality of its reporting.

This three-prong approach aligns with the major categories present in the literature, normative and biobank-specific documentation, as well as consolidates the methods into an approach that could be widely applied to population biobanks, in an attempt to harmonize the approach to authorship.

When it comes to acknowledging the biobank and its scientific leadership, this will often vary depending on the type of biobank, the data/biosamples that are available, and the effort involved in collecting and curating them [22]. For example, it is suggested that research using governmental administrative databases should not involve adding curators as co-authors because such databases are publically available [22]. On the other hand, research using hypothesis-generated or processed data/biosamples, which require time and effort from the principal investigator to prepare and curate, the possibility of co-authorship, within the spirit of collaboration, may be an appropriate consideration [22].

As discussed above, the collection, processing, and analysis of data and/or biosamples alone, is typically not considered sufficient for a granting of authorship as the ICMJE guidelines focus on substantial contributions to publications [42]. However, questions have been raised surrounding whether international guidelines on co-authorship, such as those recommended by the ICMJE, should be revised to clarify exactly what contributions would merit co-authorship [36]. Verlinden, Minssen and Huys argue that such an approach would help to “raise awareness and appreciation with regard to the more essential technical or scientific contributions,” as well as “prevent international guidelines from being increasingly ignored […]” [42]. Furthermore, alternatives have been presented in which biobank policies would provide clear guidance on how contributions can be recognized and rewarded outside of co-authorship attribution [42]. Would other solutions be deemed appropriate and sufficient by the research community and, in particular, biobank scientific leaders?

It is important to note that the ICMJE criteria for authorship attribution are cumulative. Their goal is to attribute authorship to those who deserve credit and can take responsibility for the work as a whole. It is made clear that the criteria are not intended to exclude colleagues from authorship; therefore, anyone who meets the first criterion (i.e. substantially contributes) should also be given the chance to take part in the second, third and fourth criteria, notably “the review, drafting, and final approval of the manuscript” [38].

Finally, journals should aim to develop clear authorship policies that align with and reinforce the ICMJE criteria, requiring that all four criteria be met for authorship, otherwise acknowledgment would be the appropriate credit. Although the ICMJE criteria are the current internationally accepted standard, it is important that these criteria be revised on a regular basis to ensure that they continue to reflect the needs of the scientific community while also respecting research integrity. It is also important to manage expectations for both authors and the readership of scientific journals. This can be done by improving clarity and transparency, as well as ensuring scientific integrity and fairness through appropriate credit for all authors and contributors, ultimately avoiding the inappropriate inclusion of those who do not meet the criteria. One thing is certain: credit should be given where credit is deserved, and one point that is apparent throughout is the need to have made a substantial contribution to the work, without which authorship should not be possible.

## Limitations

Some considerations for document analysis are that documents were at times found to contain insufficient detail (e.g. not all documents provided the same amount of detail or clear guidance regarding publication ethics), some were difficult to access (e.g. not all biobanks made their access documentation readily available online and required additional steps to be obtained), and for those that are made available and accessible by an organization, a selection bias may exist [26]. For example, influence by personal interpretation of publication ethics and notions of authorship may add an element of subjectivity to the elaboration of well-defined inclusion criteria. We aimed to mitigate the risk of biased inclusion by having two researchers review and assess the citations prior to inclusion in our analysis [43].

## Supporting information

S1 File Concept map outlining coding categories and sub-categories. (TIFF)

S1 Table Authorship approaches: Normative documents. (DOCX)

S2 Table Authorship approaches and mechanisms: Biobank-specific documentation. (DOCX)
