# Supplementary figures and images for: The author who wasn’t there? Fairness and attribution in publications following access to population biobanks
Source: PLoS One. 2018 Mar 23;13(3):e0194997. doi: 10.1371/journal.pone.0194997 (PMC5865744; doi:10.1371/journal.pone.0194997)

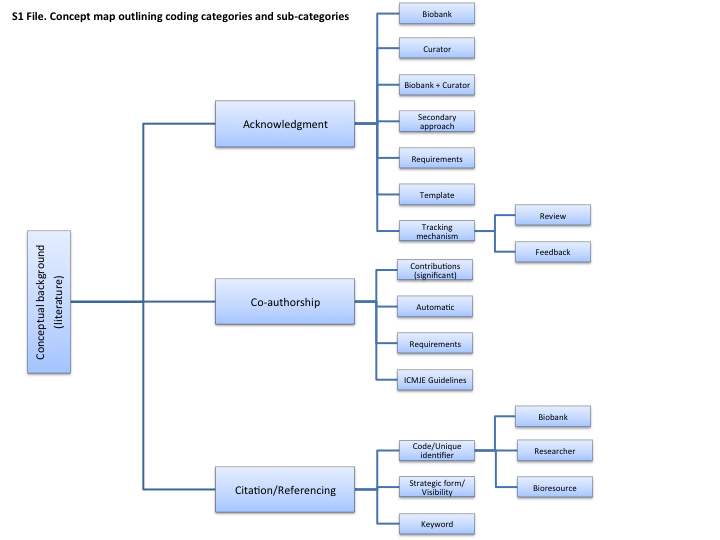

Supplement: S1 File — (TIFF) [file pone.0194997.s001.tiff]
