# Supplementary material for: The author who wasn’t there? Fairness and attribution in publications following access to population biobanks
Source: PLoS One. 2018 Mar 23;13(3):e0194997. doi: 10.1371/journal.pone.0194997 (PMC5865744; doi:10.1371/journal.pone.0194997)
Supplement: S1 Table — (DOCX) [file pone.0194997.s002.docx]

**S1 Table. Authorship approaches: normative documents**

| **Organization** | **Title** | **Scope** | **Approach** |
| --- | --- | --- | --- |
| 1. European Commission | Biobanks for Europe: A Challenge for Governance, 2012 [1] | Regional  (Europe) | **Citation**   - Custodians who have established and maintained the biobank should be rewarded. This can be accomplished by giving a unique identifier to the biobank, the researcher (ORCID ID184) or the bioresource (BRIF). |
| 2. RD-Connect | International Charter of Principles for Sharing Bio-specimens and Data, 2015 [2] | Regional  (Europe) | **Acknowledgment**   - The sharing of data and bio-specimens should follow criteria for the acknowledgment of intellectual contributions and originality through rules of authorship and intellectual property rights. |
| 3. Bioresource Research Impact Factor (BRIF) initiative | Developing a Guideline to Standardize the Citation of Bioresources in Journal Articles (CoBRA), 2015 [3] | International | **Citation**   - The most appropriate solution is to refer to bioresources in the *Methods section*. - Each individual bioresource that is actively used to perform a study should be cited as a unique digital ID (BRIF for bioresource)   E.g. Bioresource: Orphanet.  Citation: Orphanet; Le portail des maladies rares et des médicaments orphelins; Last access: April 15, 2014. [BIORESOURCE].  Discouraged approaches:   - It is discouraged to identify bioresources using the name of the bioresource rather than an ID would stem confusion. - A “bioresource field” or a specific section is not an appropriate solution because it would be too complicated and not of sufficiently broad use to warrant its creation. - Specific sentences in the *Acknowledgment section* in journal articles did not seem useful, considering the necessity of traceability and easy retrieval. |
| 4. Organisation for Economic Co-operation and Development (OECD) | OECD Guidelines on Human Biobanks and Genetic Research Databases, 2009 [4] | International | **Acknowledgment**   - In publications and presentations, researchers should acknowledge the HBGRD whose resources they have used or relied on. - The HBGRD should provide researchers with guidance on how it wishes to be acknowledged. |
| 5. Global Alliance for Genomics & Health | Framework for Responsible Sharing of Genomic and Health-Related Data, 2014 [5] | International | **Acknowledgment**   - It is appropriate to provide due credit and acknowledgment of all who contributed to the results.   * It is important to track the chain of data access and/or exchange to its source |
| 6. International Society for Biological and Environmental Repositories (ISBER) | Best Practices for Repositories: Collection, Storage, Retrieval and Distribution of Biological Materials for Research, 2012 [6] | International | **Acknowledgment**   - In publications that result from the use of specimens, the repository should be acknowledged as the source of the specimens. - When repository staff members actively participate in the research project itself (e.g., by providing substantial intellectual input beyond the routine role of the repository, which may include data analysis or manuscript preparation), they should be considered co-authors. |
| 7. Italian Society of Human Genetics | Guidelines for Genetic Biobanks, 2004 [7] | National  (Italy) | **Citation**   - In the event of publication of results obtained on material stored in the biobank, the investigators must promise to cite the origin of the sample and to quote the biobank in the acknowledgments of any scientific production.   * The authors must send a copy of the publication to the biobank.  * No sample will be sent without the signed request form. |
| 8. Cancer Research UK, Economic & Social Research Council (ESRC), Medical Research Council (MRC) and Wellcome Trust | EAGDA Report: Governance of Data Access, 2015 [8] | National  (United Kingdom) | **Acknowledgment**   - The appropriate solution is to ensure that the rights of those who worked to produce the data are appropriately acknowledged and respected in publications from secondary users.   * Co-authorship should recognize significant contributions to a publication and not be a default requirement for permitting access to data. |
| 9. Medical Research Council (MRC) and Wellcome Trust | Access to Collections of Data and Materials for Health Research: A Report to the Medical Research Council and the Wellcome Trust, 2006 [9] | National  (United Kingdom) | **Acknowledgment**   - The contributions of the resource and its curators must always be acknowledged in publications.   - This is usually expressed in standard acknowledgment notes at the beginning or end of articles |
| 10. National Cancer Research Institute (NCRI) & Confederation of Cancer Biobanks (CCB) | Biobank Quality Standard – Collecting, storing and providing human biological material and data for research, 2014 [10] | National  (United Kingdom) | **Acknowledgment**   - Researchers should be required to acknowledge the biobank as the source of the samples used in their research. |
| 11. National Institutes of Health (NIH) | NIH Genomic Data Sharing Policy, 2014 [11] | National  (United Kingdom) | **Acknowledgment**   - Investigators using controlled-access and unrestricted-access data from NIH-designated data repositories must acknowledge in all oral or written presentations, disclosures, or publications the specific dataset(s) and applicable accession number(s), and the NIH-designated data repositories. |

**References for S1 Table**

1. European Commission. Biobanks for Europe: a challenge for governance. Brussels: European Commission; 2012.
2. Mascalzoni D, Dove ES, Rubinstein Y, Dawkins HJ, Kole A, McCormack P, et al. International Charter of principles for sharing bio-specimens and data. Eur J Hum Genet; 2015;23(6): 721-728.
3. Bravo E, Calzolari A, De Castro P, Mabile L, Napolitani F, Rossi AM, et al. Developing a guideline to standardize the citation of bioresources in journal articles (CoBRA). BMC Medicine. 2015;13(33): 1-12.
4. Organization for Economic Co-operation and Development. OECD guidelines on human biobanks and genetic research databases. Paris: OECD; 2009
5. Global Alliance for Genomics and Health. Framework for responsible sharing of genomic and health-related data. Toronto: Global Alliance for Genomics and Health; 2014.
6. International Society for Biological and Environmental Repositories. Best practices for repositories: collection, storage, retrieval and distribution of biological materials for research. 3rd ed. Vancouver: ISBER; 2012.
7. Italian Society of Human Genetics. Guidelines for Genetic Biobanks. Italian Society of Human Genetics; 2004.
8. Cancer Research UK, Economic & Social Research Council, Medical Research Council, Wellcome Trust. EAGDA Report: Governance of Data Access. London: Wellcome Trust; 2015.
9. Lowrance WW. Access to collections of data and materials for health research: a report to the Medical Research Council and the Wellcome Trust. London: Wellcome Trust; 2006.
10. National Cancer Research Institute, Confederation of Cancer Biobanks. Biobank Quality standard: collecting, storing and providing human biological material and data for research, London: NCRI; 2014.
11. National Institutes of Health. NIH Genomic Data Sharing Policy. Bethesda (MD): NIH; 2014.
