# Supplementary material for: The author who wasn’t there? Fairness and attribution in publications following access to population biobanks
Source: PLoS One. 2018 Mar 23;13(3):e0194997. doi: 10.1371/journal.pone.0194997 (PMC5865744; doi:10.1371/journal.pone.0194997)
Supplement: S2 Table — (DOCX) [file pone.0194997.s003.docx]

**S2 Table. Authorship approaches and mechanisms: biobank-specific documentation**

| **Biobank** | **Title** | **Country** | **Approach** | **Mechanism** |
| --- | --- | --- | --- | --- |
| 1. Biobank Graz Medical University | *Material Transfer Agreement of Biobank Graz*, 2016 [1] | Austria | **Acknowledgment**  (source of the materials)  (standard sentence) | Proposed publications must be disclosed to the Medical University of Graz. |
| 2. Canadian Health Measures Survey (CHMS) Biobank | *Access Requirements and Protocols for the Canadian Health Measures Survey Biobank*, November 2016 [2] | Canada | **Acknowledgment**  (CHMS program, Statistics Canada and participants)  (standard sentence) | Suggest discussing analysis and interpretation of results with the CHMS program at Statistics Canada prior to publishing.  Must attempt to publish within one year of receiving the data or justify the need for an extension.  Copy of publications must be provided to Statistics Canada. |
| 3. Canadian Longitudinal Study on Aging (CLSA) | CLSA, *Data and Sample Access Policy and Guiding Principles*, September 2014 [3]  *CLSA Access Agreement*, April 2016 [4] | Canada | **Acknowledgment**  (source of CLSA data and biosamples)  Must reference:   - Source of funding for CLSA and its data platform; and - Core CLSA team responsible for creation and implementation of platform.   (standard paragraph) | Proposed presentations or publications must be provided to McMaster University for review at least 10 days prior to submission.  Review limited to ensuring that:   - Participants cannot be identified; and - Results presented in scientifically accurate manner to prevent the stigmatization of participants and of the communities they belong to. |
| 4. Canadian Partnership for Tomorrow Project (CPTP) | CPTP, *Access Policy*, May 2016 [5]  *CPTP Publications Policy*, October 2015 [6] | Canada | **Co-authorship**  Must offer to one or more members of each of the Cohorts whose data and/or biosamples are used  (possibility for additional active contributors to CPTP, as appropriate)  * Compliance with ICMJE Guidelines for authorship (recognized standard).  **Acknowledgment**  (contribution of CPTP, the participating cohorts and active contributors, where applicable)  (standard sentence) | Proposed publications and abstracts must be submitted to the Access Office upon submission for publication to the journal or to a conference.  All presentations should be submitted to the Access Office one week prior to the presentation.  Review limited to ensuring that:   - CPTP, its participating cohorts without a representative co-author, and any active contributors are properly acknowledged; - No individuals or communities are identified; and - Analyses included are within the scope of the Approved Research Project as per the *CPTP Data and Material Access Agreement* and the *CPTP Data and Material Access Application Form*. |
| 5. China Kadoorie Biobank (CKB) | *CKB Data Access and Sample Preservation Policy*, v.1 [7]  *Data Access Agreement relating to the China Kadoorie Biobank*, v.2, August 2015 [8] | China | **Acknowledgment**  (biobank)  (standard paragraph)  + Link to reference search tools, such as PubMed and MEDLINE (when possible)  **Possibility of Co-authorship**  (if appropriate)  + List of co-authors should generally end with “on behalf of the China Kadoorie Biobank collaborative group” (unless decided otherwise).  * Compliance with ICMJE Guidelines for authorship (recognized standard). | Proposed publications must be provided to:   - Steering Committee at least 28 days prior to submission; and - University at least 30 days prior to submission for review and comments.   All publications to be made available from PubMed Central and Europe PubMed Central within six months of publication. |
| 6. EORTC Prospective Tissue & Biofluid Collection | *Human Biological Material Collection, Storage and Use*, v.2.1, June 2015 [9]  *Disclosure of Results and Publication Policy*, v.4.2, March 2015 [10] | Belgium | **Acknowledgment**  (source of the materials)  + ‘EORTC’ must be visible in the publication’s header (for clinical study results and ancillary research to EORTC protocols conducted by EORTC members)  Types of publication:   - Joint publication (1^st^) - Site-specific publication (2^nd^), possible only:   1. After joint publication;   2. After expiration of the 12-month database lock;   3. If joint study is terminated.   **Possibility of Co-authorship**  (if appropriate)  Responsibilities of the 1^st^ author (usually the Study Coordinator):   - Write the first draft of the manuscript (within 6 months of receiving the analysis produced by EORTC Headquarters); - Ensure all authors see and approve the final manuscript.   * Compliance with ICMJE Guidelines for authorship (i.e. substantial contribution to the work and able to take responsibility for the content), otherwise acknowledgment. | Site-specific publications:  Proposed publications must be provided to the Head of the EORTC Statistics Department at least 30 days prior to submission for review and comment. |
| 7. CONSTANCES Cohort | *Constances Charter (draft)*, January 2017 [11] | France | **Co-authorship**  At least one member of the CONSTANCES team must appear in any publication stemming from the use of their data (very substantial scientific and technical activity).  **Acknowledgment**  (source of the data, funder, possible nominative acknowledgments of individual researchers and other partners)  (standard paragraph)  + ‘CONSTANCES’ must appear in the title of all publications.  + All publications must refer to at least one of the methodological articles published by the CONSTANCES team. | Proposed publication must be provided to the CONSTANCES Director upon submission. |
| 8. Northern Ireland Biobank (NIB) | *NIB Access Policy*, v.1, February 2013 [12] | Ireland | **Acknowledgment**  (source of the biosamples)  *Further conditions are listed in the Terms and Conditions in the preliminary application form [not available online]. | Proposed publications must be provided to the NIB. |
| 9. Telethon Network of Genetic Biobanks (TNGB) | *Guidelines for Genetic Biobanks*, May 2004 [13]  *Telethon Network of Genetic Biobanks (TNGB) Charter*, December 2015 [14] | Italy | **Acknowledgment**  (source of the biosamples, service provided by the biobank)  **Possibility of Co-authorship** (when appropriate)  + If TNGB staff actively takes part in the activities that are outlined in the publication, then co-authorship may be admissible. | Copy of accepted publications must be provided to the biobank. |
| 10. Cohort of Norway (CONOR) | *Guidelines for access to CONOR materials*, December 2004 [15] | Norway | **Acknowledgment**  (source of the data and CONOR website)  (standard sentence)  + ‘Cohort of Norway’ or ‘CONOR’ must be mentioned in the title *or* abstract of the publication for visibility in PubMed searches.  + Reference to the biobank’s methodological article. | Proposed publications must be provided to CONOR secretariat prior to submission. Comments must be returned within 14 days.  Copy of accepted publications must be provided to the biobank. |
| 11. Nord-Trøndelag Health Study (The HUNT Study) | HUNT, *Guidelines for publication of research results using HUNT-data*, 2016 [16]  HUNT, *Guidelines for administration and use of research data from the Nord-Trøndelag Health Study (HUNT)*, Document ID 1927, v.1.1 [17] | Norway | **Acknowledgment**  (contribution of HUNT and the institutes that have helped with data collection)   - Solely HUNT data:   “HUNT Study” must be part of the title (if no objection from journal);   - HUNT data + other data sources:   HUNT to be mentioned in the title (when possible) or in the Methods section;  (standard paragraph)  + ‘HUNT’ as a keyword.  * Vancouver rules of authorship must be followed for authorship | Proposed abstracts, presentations or publications must be provided to the Publication Committee prior to submission (not a review committee for content).  Review limited to ensuring that:   - Publication is in line with the publication plan; and - Authors have respected the co-authorship rules and the agreement on the use of variables. Typical processing time is between 1-2 weeks (not delay publication process). |
| 12. Norwegian Mother and Child Cohort Study (MoBa) | *MoBa Guidelines for Research*, October 2015 [18] | Norway | **Acknowledgment**  (standard paragraph)  + Identify MoBa in the Methods section.  + Include MoBa and NIPH logos on any posters or presentations (scientific credit). | Proposed publications must be provided to MoBa prior to submission (administrative review). Review will be conducted within two weeks of confirmed receipt.  Approval from MoBa is not required for abstracts and posters; however, a copy must be provided to MoBa.  Review limited to ensuring that:   - MoBa is correctly described; - Mandatory references are included; - Analyses align with the scientific aims of the approved application. |
| 13. LifeGene | *LifeGene Access and IP Policy*, v.2.0, February 2015 [19]  *Material and Data Transfer Agreement* [20] | Sweden | **Acknowledgment**  (source of data/biosamples and funding)  (clear guidance provided by LifeGene)  **Co-authorship** (when appropriate) | Proposed publications must be submitted to a journal within 6 months of completing a study.  Review of proposed publications by LifeGene is not required prior to submission; however, a copy should still be provided. |
| 14. 1958 British Birth Cohort Study | *Policy for use and oversight of samples and data arising from the Biomedical Resource of the 1958 Birth Cohort (National Child Development Study)*, v.5, January 2015 [21] | United Kingdom | **Acknowledgement**  (data creators, depositors or copyright holders, service funders, data providers and data collections used) | Proposed publication to be provided prior to submission (only if controversial).  Provide bibliographic details of any publication. |
| 15. Born in Bradford (BiB) | *Guidance and conditions for collaborators on the Born in Bradford programme*, n.n. [22] | United Kingdom | **Acknowledgment**  (Bradford community)  (standard paragraph)  + Encourage inclusion of ‘Born in Bradford’ in publication’s title (easily identifiable) *or* as a keyword and in the abstract.  + Reference to the methodological article within the Methods section.  **Contributorship** (if authorship criteria not met)  (see BMJ guidelines)  **Possibility of Co-authorship**  (if appropriate)  * Compliance with ICMJE Guidelines for authorship (recognized standard). | Proposed abstracts and publications must be provided to the BiB Executive Group prior to submission for review (to be conducted within two weeks of receipt).  Review is limited to ensuring that:   - Confidentiality is protected; - Study not brought into disrepute; - No overlap with other papers.   BiB may also provide advice and feedback, when deemed useful.  A copy of accepted publications to be provided to the BiB Executive Group and the Parent Governors group. |
| 16. Generation Scotland (GS) | *Section 5.0* and *Appendix 11* of the *Generation Scotland Management, Access and Publications Policy*, June 2016 [23] | United Kingdom | **Co-authorship**  The *Publication Policy* applies to:   1. Internal publications: members of GS Executive Committee & Expert Working Groups   (e.g. research papers based on the resource or papers describing the biobank and its work)   1. External publications: collaborators accessing the GS resource   (e.g. original papers by researchers who have received access to GS)  Criteria (all must be met):   - ‘Academic contribution’ to study design, data collection and processing, analysis and/or reporting (both intellectual responsibility and substantive work); - Critical revision of article; and - Ability to defend the paper as a whole.   + Expectation that GS be included as a co-author on abstracts and that the resource be referenced and acknowledged.  **Acknowledgment**  If criteria are not met or an exception exists – full acknowledgment of GS  (standard text, including funding organizations)  + ‘Generation Scotland’ as a keyword. | All publications must be approved by GS prior to submission to a journal.  Review entails ensuring that:   - Confidentiality is protected; - Any patentable results are identified; - Contribution of GS is recognized; - Paper will not bring the Project or Future Projects into disrepute; - Study does not overlap with other papers published or in preparation; and - GS requirements are adhered to (Appendix 11 *Policy*).   GS may also provide advice and feedback, when deemed useful (not formal peer review).  Copy of published abstracts and presentations (conferences and scientific meetings) – to be provided to GS (no need to approve abstracts prior to submission).  Collaborators to send copies of the submitted version of a publication and to inform GS when paper is accepted (send electronic version). |
| 17. Million Women Study | *Data Access and Sharing Policy for the Million Women Study*, September 2015 [24]  *Million Women Study*  *Data Transfer Agreement*, September 2015 [25] | United Kingdom | **Co-authorship**  + Include data contributor as an author on any publication.  + Million Women Study team and individual investigators, if appropriate, must be named as co-authors (recognize contributions by past and current staff/collaborators).  **Acknowledgment**  (source of the data – University and the study)  + Inclusion of ‘Million Women Study’ in the title, subheading *or* abstract of the publication, when possible. | Proposed publications must be provided to the study investigators at least 28 days prior to submission for their consideration.  Copy of accepted publications to be provided to the University. |
| 18. UK Biobank | *Access Procedures: Applications and review procedures for access to the UK Biobank Resource*, v.1.0, November 2011 [26]  *Publication of findings – Guidance* [27] (website) | United Kingdom | **Acknowledgment**  (template sentence)  + Link to reference search tools, such as PubMed and MEDLINE (when possible) | Applicant PIs must do their best to publish findings within six months of the agreed upon date of completion of the study (C11.1).  Approval is not needed by UK Biobank. However, the applicant must provide all publications at least two weeks before their expected date of first public presentation or publication in any format (e.g. abstract, presentation, paper) (C11.2).  (not require future notifications of the same work)  Applicant must advise UK Biobank in advance if publication is likely to provoke controversy or attract significant public attention (C11.2). |
| 19. Avon Longitudinal Study of Parents and Children (ALSPAC) | *Checklist for Papers on the Avon Longitudinal Study of Parents and Children (ALSPAC)*, v.25, August 17, 2016 [28]  *Access policy*, v.7.0, September 2016 [29] | United Kingdom | **Acknowledgment**  (template paragraph)   - ALSPAC - Any specific grant funding for particular data that was used   + Standard statement on the data dictionary in the Methods section and reference to the webpage.  + ‘ALSPAC’ as a keyword. | All publications must be provided to the ALSPAC Executive (for approval), along with a signed and completed papers checklist prior to submission (no need to submit conference abstracts). All papers will be processed within two weeks of receipt.  Review limited to ensuring that:   - Confidentiality is protected; and - Paper will not bring the study into disrepute.   Executive reserves right to withhold any paper that could potentially breach confidentiality from publication (will work with authors to overcome).  Executive also provides advice and feedback, when deemed useful (not formal peer review). |
| 20. Newcastle Biomedicine Biobank | *Access Policy*, v.2.0, January 2013 [30] | United Kingdom | **Acknowledgment**  (standard paragraph) | Proposed publications must be provided to the custodian prior to submission to establish compliance with the terms of acceptance.  Publications are to be deposited in the UK PubMed Central database within three months of publication. |
| 21. AMGEN | *Amgen Guidelines for Publications* (website) [31] | United States of America | **Acknowledgement**  (financial support, technical assistance and contributions from those not meeting the authorship criteria)  **Possibility of Co-authorship**  (where appropriate)  + Guest authorship, ghost writing or plagiarism are prohibited.  * Compliance with the ICMJE guidelines for authorship attribution (substantive input throughout). | No mechanism mentioned. |
| 22. Growing Up Today Study (GUTS) | *Guidelines for use of the Growing Up Today Study: External Collaborators*, April 2012 [32] | United States of America | **Co-authorship**  + At least one member of the GUTS team may be a co-author on publications stemming from collaborations, and must review and approve the publication/presentation prior to submission (in writing). The same applies for posters and presentations. | Proposed publications must be provided to the Channing Laboratory and the Department of Medicine at the Brigham and Women’s Hospital prior to submission for review (review will take at least four weeks). |
| 23. Marshfield Clinic Personalized Medicine Research Project (PMRP) | *Data and Tissue Access Guidelines*, July 2010 [33] | United States of America | **Co-authorship**  + At least one Marshfield Clinic investigator should be offered co-authorship.  **Acknowledgement**  (support from the Clinical and Translational Science Award (CTSA))  + Reference to the biobank’s methodological article. | No prior approval of proposed publications is required by PMRP. |
| 24. Nurses’ Health Study (NHS) | *Guidelines for External Collaborators: Use of the Nurses’ Health Studies Archived Data*, n.n. [34] | United States of America | **Co-authorship**  + At least one member of the NHS team will be a co-author on publications stemming from collaborations, and must review and approve the publication prior to submission. | Results must be presented at an NHS study meeting prior to submission for publication.  Proposed publications must be provided to Dr. Meir Stampfer, Co-Director of the Channing, and the Department of Medicine at the Brigham and Women’s Hospital prior to submission for approval. |
| 25. The European Prospective Investigation into Cancer and Nutrition (EPIC) Biobank | EPIC, *The EPIC Access Policy*, 2014 [35]  EPIC, *Publication guidelines for EPIC related studies*, January 2014 [36] | National (Europe) | **Co-authorship**  (automatic based on specific rules)   1. Papers reporting on EPIC materials/data ALONE.    1. *Writing group members* (5-7)    2. *Representatives of individual centres* (grouped by country, in alphabetical order of countries, according to the agreed number of authors per centre)    3. *IARC, ICL and senior author* (if applicable) 2. Consortium based papers (materials/data from several studies - including EPIC) 3. *Potential writing groups members* 4. *EPIC representatives*: up to 12 authors (1 per country + 1 author from IARC + 1 author Imperial College centres)   * If country has not contributed data, collaborators from that centre will not be included in the author list | All publications must be approved by EPIC Steering Committee members before submission. This entails:   - Preparing and agreeing within the writing group; and - Circulating within Steering Committee with request for approval + assignment of co-author representatives from EPIC centres (with their comments)   Co-authors should have at least two weeks to comment on the manuscript. |

**References for S2 Table**

Medical University of Graz. Biobanking and biospecimen research. 2015. Medical University of Graz [Internet]. Available from: http://biobank.medunigraz.at/fileadmin/forschen/biobank/pdf/FB328en_Biobank_Graz_Material_Transfer_Agreement.pdf.

Statistics Canada. Access requirements and protocols for the Canadian health measures survey biobank. 2017 [cited 6 July 2017]. Statistics Canada [Internet]. Ottawa: Statistics Canada. Available from: http://www.statcan.gc.ca/eng/survey/household/5071y.

Canadian Longitudinal Study on Aging. Data and Sample Access Policy and Guiding Principles. 2014. McMaster University [Internet]. Available from: https://clsa-elcv.ca/doc/1039.

Canadian Longitudinal Study on Aging. CLSA Access Agreement. 2016. McMaster University [Internet]. Available from: https://clsa-elcv.ca/doc/1042.

Canadian Partnership Against Cancer, Canadian Partnership for Tomorrow Project. Access Policy. 2016. [Internet]. Available from: https://portal.partnershipfortomorrow.ca/sites/portal-live-7.x-5.10-020320171455--partnershipfortomorrow.ca/files/Access_Policy_Approved_May_11_final.pdf.

Canadian Partnership Against Cancer, Canadian Partnership for Tomorrow Project. CPTP Publications policy. 2015. [Internet]. Available from: https://portal.partnershipfortomorrow.ca/sites/portal-live-7.x-5.10-020320171455--partnershipfortomorrow.ca/files/CPTP%20Publications%20Policy%20-%20Approved%20Oct%2022%202015.pdf.

China Kadoorie Biobank. CKB data access and sample preservation policy, v1. 2014. [Internet]. Available from: http://www.ckbiobank.org/site/binaries/content/assets/resources/pdf/ckb-data-access-policy_1april2014.pdf.

China Kadoorie Biobank. Data access agreement relating to the China Kadoorie Biobank, v2. 2015. University of Oxford [Internet]. Available from: http://www.ckbiobank.org/site/binaries/content/assets/resources/pdf/ckb-data-access-agreement-template.pdf.

EORTC Prospective Tissue & Biofluid Collection. Human Biological Material Collection, Storage and Use, v2.1. 2015 [Internet]. Available from: http://www.eortc.org/app/uploads/2017/03/POL020-v-2.1-NS-1.pdf.

EORTC Prospective Tissue & Biofluid Collection. Disclosure of Results and Publication Policy, v4.2. 2015. [Internet]. Available from: http://www.eortc.org/app/uploads/2017/03/POL009-v-4.2-NS.pdf.

CONSTANCES Cohort. Constances Charter (draft), 2017. [Internet]. Available from: http://www.constances.fr/charter.

Northern Ireland Biobank. Access Policy. 2013. [Internet]. Available from: http://www.nibiobank.org/documents/nib-access-policy-version-1-270213.pdf.

Italian Society of Human Genetics. Guidelines for Genetic Biobanks. 2004. [Internet]. Available from: http://biobanknetwork.telethon.it/Document/DownloadFile/19.

Italian Society of Human Genetics. Telethon Network of Genetic Biobanks Charter. 2015. [Internet]. Available from: http://biobanknetwork.telethon.it/Document/DownloadFile/22.

Cohort of Norway. Guidelines for access to CONOR materials. 2015. [Internet]. Available from: http://bbmri-lpc.iarc.fr/mica/sites/default/files/AccessGuidelines.pdf.

HUNT Research Centre. Guidelines for publication of research results using HUNT-data. 2016. [Internet]. Available from: https://www.ntnu.edu/documents/140075/1268289603/2016_Guidelines+for+publication+of+research+results+using+HUNT.pdf/574455bd-4b71-4ebd-aee8-1c8f4683ceef.

HUNT Research Centre. Guidelines for administration and use of research data from the Nord-Trondela Health Study. [Internet]. Available from: https://www.ntnu.edu/documents/140075/0/Guidelines_for_the_use_of_HUNT_data.pdf/1a597987-8149-4a5f-a427-d2435ade310e.

Norwegian Mother and Child Cohort Study. Guidelines for research. 2015. [Internet]. Available from: https://www.fhi.no/globalassets/dokumenterfiler/retningslinjer-moba-eng.pdf.

LifeGene. LifeGene Access and IP Policy, v2.0. 2015. [Internet] Available from: https://www.lifegene.se/PageFiles/591/LifeGene%20Access%20and%20IP%20Policy%202015.pdf.

LifeGene. Material and Data Transfer Agreement. [Internet] Available from: https://www.lifegene.se/For-scientists/Call-for-proposal/.

1958 British Birth Cohort Study. Policy for use and oversight of samples and data arising from the biomedical resource of the 1958 Birth Cohort (National Child Development Study), v5. 2015. [Internet]. Available from: http://www.metadac.ac.uk/files/2016/04/1958bc-POLICY-DOCUMENT-v5-Jan-2015.pdf.

Born in Bradford. Guidance and conditions for collaborators on the Born in Bradford program. [cited 6 July 2017]. Born in Bradford. [Internet]. Available from: https://borninbradford.nhs.uk/research/guidance-for-collaborators/.

Generation Scotland. Management, access and publications policy. 2016. [Internet]. Available from: http://www.edinburgh.ac.uk/files/atoms/files/gsmapp_access_policy_v6-7_december_2016_final_0.pdf.

Nuffield Department of Population Health Cancer Epidemiology Unit. Data Access and Sharing Policy for the Million Women Study, v1.2. 2016. University of Oxford [Internet]. Available from: http://www.millionwomenstudy.org/files/MWS-DataAccessPolicy.pdf.

Nuffield Department of Population Health Medical Sciences Division. Million Women Study Data transfer agreement. 2015. University of Oxford [Internet]. Available from: http://www.millionwomenstudy.org/files/MWS-DataTransferAgreement.docx.

UK Biobank. Access procedures: Application and review procedures for access to the UK Biobank resource. 2011. [Internet]. Available from: http://www.ukbiobank.ac.uk/wp-content/uploads/2011/11/Access_Procedures_Nov_2011.pdf.

UK Biobank. Publications of findings – guidance. 2016 [cited 6 July 2017]. UK Biobank. [Internet]. Available from: http://www.ukbiobank.ac.uk/publication-of-findings-guidance/.

Avon Longitudinal Study of Parents and Children. Checklist for papers on the Avon Longitudinal Study of Parents and Children (ALSPAC), v26. 2016. [Internet]. Available from: http://www.bristol.ac.uk/media-library/sites/alspac/documents/researchers/ALSPAC-publications-checklist.pdf.

Avon Longitudinal Study of Parents and Children. Access Policy, v7. 2016. [Internet]. Available from: http://www.bristol.ac.uk/media-library/sites/alspac/documents/ALSPAC_access_policy.pdf.

Newcastle Biomedicine Biobank. Access policy, v2. 2013. [Internet]. Available from: http://www.ncl.ac.uk/media/wwwnclacuk/newcastlebiobank/files/nbrtb-access-policy.pdf.

AMGEN. AMGEN guidelines for publications. 2017 [cited 6 July 2017]. AMGEN [Internet]. Available from: http://www.amgen.com/about/how-we-operate/policies-practices-and-disclosures/ethical-research/amgen-guidelines-for-publications/.

Growing Up Today Study. Guidelines for use of the Growing Up Today Study: External Collaborators. 2012. [Internet]. Available from: http://www.gutsweb.org/images/PDFs/guts-data-use.pdf.

Marshfield Clinic Personalized Medicine Research Project. Data and tissue access guidelines. 2010. [Internet]. Available from: https://www.marshfieldresearch.org/Media/Default/CHG/PMRP%20Forms/MCRF-Centers-PMRP-TissueAccessGuidelines1.1.pdf.

Nurses’ Health Study. Guidelines for external collaborators: Use of the Nurses’ Health Studies archived data. [Internet]. Available from: http://www.nurseshealthstudy.org/sites/default/files/pdfs/Guidelines_Archived%20Data.pdf.

World Health Organization - International Agency for Research on Cancer. The European Prospective Investigation in Cancer and Nutrition (EPIC) study: The EPIC access policy. 2014. [Internet]. Available from: http://epic.iarc.fr/docs/EPIC_Access_Policy_and_Guidelines.pdf.

World Health Organization - International Agency for Research on Cancer. Publication guidelines for EPIC related studies, v3. 2014. [Internet]. Available from: http://epic.iarc.fr/docs/EPIC_Publication%20Guidelines.pdf.
